# Mutations of IFT81, encoding an IFT-B core protein, as a rare cause of a ciliopathy

**DOI:** 10.1186/2046-2530-4-S1-P7

**Published:** 2015-07-13

**Authors:** I Perrault, J Halbritter, J Porath, X Gerard, D Braun, H Gee, H Fathy, S Saunier, V Cormier-Daire, S Thomas, T Attié-Bitach, N Boddaert, M Taschner, M Schueler, E Lorentzen, R Lifton, E Otto, P Bastin, J Kaplan, F Hildebrandt, JM Rozet

**Affiliations:** 1 Laboratory of Genetics in Ophthalmology, Paris, France; 2 Boston Children's Hospital, Division of Nephrology, Boston, MA, USA; 3 University of Alexandria, Pediatric Nephrology Unit, Alexandria, Egypt; 4 Molecular Bases of Hereditary Kidney Diseases, INSERM UMR 1163, Paris, France; 5 Molecular and Physiopathological Bases of Osteochondrodysplasia, Paris, France; 6 Embryology and Genetics of Human Malformation, Paris, France; 7 Department of Pediatric Radiology, Hôpital Necker-Enfants Malades, Paris, France; 8 Department of Structural Cell Biology, Max Planck Institute of Biochemistry, Martinsried, Germany; 9 Department of Genetics and Howard Hughes Medical Institute, Yale University School of Medicine, New Haven, CT, USA; 10 Departments of Pediatrics, University of Michigan, Ann Arbor, MI, USA; 11 Institut Pasteur and CNRS, Trypanosome Cell Biology Unit, Paris, France

## Objective

To identify ciliopathy-causing genes in a very large cohort of patients with symptoms consistent with cilia dysfunction.

## Methods

1,056 index cases with nephronophthisis-related ciliopathies were screened for mutations in all genes encoding components of IFT-B and 572 unrelated individuals with early onset retinal dystrophies or multisystemic ciliopathies were subjected to targeted ciliome resequencing.

## Results

Homozygosity for *IFT81 *mutations were identified in two consanguineous sporadic cases. The first individual harbored a splice site change predicted to result in an inframe exon skipping; the second carried a 4 bp deletion resulting in a loss-of-stop with extension of the deduced protein by 10 amino acids. The spectrum of *IFT81*-related disease expression included nephronophthisis, retinal dystrophy, cerebellar atrophy, and polydactyly. Fibroblasts from one affected individual showed no difference to control cells with regard to IFT81 localization or binding to IFT25, but a statistically significant decrease in ciliated cell abundance was noted. GLI2 expression and ciliary localization were impaired suggesting altered sonic hedgehog signaling.

## Discussion and conclusion

Mutations in all components of IFT-A complex have been reported to cause ciliopathy phenotypes. In contrast, only two peripheral IFT-B members, IFT172 and IFT80, were known to be involved in these conditions. The identification of mutations in the IFT-B core protein IFT81 in two unrelated patients out of 1268 individuals with ciliopathy further elucidate the role of this complex in human disease and show that defects in the IFT-B core are an exceedingly rare finding supporting the view that it is indispensable for ciliary assembly in development.

